# Characterization, Antimicrobial Effects, and Cytocompatibility of a Root Canal Sealer Produced by Pozzolan Reaction between Calcium Hydroxide and Silica

**DOI:** 10.3390/ma14112863

**Published:** 2021-05-27

**Authors:** Mi-Ah Kim, Vinicius Rosa, Prasanna Neelakantan, Yun-Chan Hwang, Kyung-San Min

**Affiliations:** 1 Department of Conservative Dentistry, School of Dentistry and Institute of Oral Bioscience, Jeonbuk National University, Jeonju 54896, Korea; miah2018@hanmail.net; 2 Discipline of Oral Sciences, Faculty of Dentistry, National University of Singapore, Singapore 119085, Singapore; denvr@nus.edu.sg; 3 Discipline of Endodontology, Department of Restorative Dental Sciences, Faculty of Dentistry, The University of Hong Kong, Hong Kong 999077, China; prasanna@hku.hk; 4 Department of Conservative Dentistry, School of Dentistry, Chonnam National University, Gwangju 61186, Korea; ychwang@chonnam.ac.kr; 5 Research Institute of Clinical Medicine of Jeonbuk National University, Jeonju 54907, Korea; 6 Biomedical Research Institute of Jeonbuk National University Hospital, Jeonju 54907, Korea

**Keywords:** calcium hydroxide, silicate, pozzolan, root canal, sealer

## Abstract

This study aimed to evaluate a newly developed pozzolan-based bioceramic sealer (PZBS) regarding setting time, radiopacity, antibacterial effect, and cytocompatibility. The PZBS was manufactured by mixing calcium hydroxide and silica. The pozzolan reaction was verified by identification of calcium silicate hydrate (C-S-H) using X-ray diffraction analysis. The initial setting time and radiopacity were measured using the ISO 6876/2012 protocol in comparison with other commercially available calcium silicate (CS) sealers. The antibacterial effect of PZBS on biofilms cultured in the bovine root canal was evaluated by measurement of colony-forming units and volume of biofilms in comparison with other calcium hydroxide pastes. The morphological features of the biofilms were observed by scanning electron microscopy (SEM). The cytocompatibility of PZBS was assessed by the viability of bone marrow–derived mesenchymal stem cells and scratch wound healing rate in comparison with other CS sealers. The morphology of the cells cultured on the tested sealers was observed by SEM. The detection of the CS peak confirmed the formation of C-S-H. The initial setting time of PZBS was around 11 h, which was twice as long as the other tested sealers. The radiopacity of PZBS was 4.3 mm/Al, which satisfied the ISO criteria. The antibacterial effect and cytocompatibility of PZBS were comparable to those of the commercially available intracanal medicaments and CS endodontic sealers, respectively. The PZBS has the potential to be used for root canal obturation, and is expected to exert a favorable antibacterial effect.

## 1. Introduction

In recent years, mineral trioxide aggregate (MTA)-based root canal sealers have gained attention because of their favorable biocompatibility and physical properties [1]. MTA is basically Portland cement, which was invented by an English bricklayer, Joseph Aspdin, early in the 19th century, and the reaction between Portland cement and water produces calcium silicate hydrate (C-S-H) [2]. Similarly, calcium silicate (CS), which is the main constituent of MTA-based sealer, sets by absorbing ambient moisture present in the root canal [1]. Therefore, slight moisture in root canal dentin may be advantageous to sealing, but resin-based sealer is affected by it [3]. Furthermore, several studies showed that MTA-based sealers have higher biocompatibility compared to resin-based sealers [4,5,6]. However, it is generally known that MTA has limited antimicrobial effects against some microorganisms [7]. In this respect, before canal obturation, clinicians use intracanal medicaments with an expected antibacterial effect, such as calcium hydroxide [Ca(OH)_2_]. The intracanal medicaments should be removed completely from the root canal system since the remaining material can play negative roles in the prognosis of endodontic treatment [8]. Moreover, the removal of the intracanal medicaments from the complex root canal system is difficult and time-consuming.

Before Portland cement was developed, pozzolan cement was extensively used in ancient architecture such as the Colosseum in Rome [9]. The Greeks and Romans used calcined limestone and later developed the pozzolanic cement by grinding together lime and volcanic ash called “pozzolan”, which was first found near Port Pozzuoli, Italy [9]. By modern definition, a pozzolan is “a siliceous material that chemically reacts with Ca(OH)_2_ to form compounds having cementitious properties”. In chemical terms, the pozzolan reaction occurs between Ca(OH)_2_ and silica. The pozzolan reaction also produces the same hydration product (C-S-H) as cement, following the formula:

3[Ca(OH)_2_] + 2[SiO_2_] = [3(CaO)·2(SiO_2_)·3(H_2_O)]

The pozzolan reaction occurs over a much longer time scale than Portland cement, and Ca(OH)_2_ remains present until the setting occurs [10]. Therefore, pozzolan-based root canal filling material can exert an antibacterial effect for a considerable time before setting, and is beneficial for eradicating the bacteria in the canal system. In the present study, we developed a root canal sealer based on the pozzolan reaction, which can be used with the single gutta-percha cone technique. In addition, the Ca(OH)_2_ powder was nanosized with the goal of achieving an improved antibacterial effect and facilitating the setting reaction of the material. Indeed, this is the first attempt to use the pozzolan reaction in the root canal to induce the formation of C-S-H, the final product for root canal filling material. Furthermore, the sealer was expected to have a potent antibacterial effect during the setting due to the nanosized Ca(OH)_2_. In other words, it is developed to be used both for intracanal medicament and root canal filling material. However, there is no information regarding the material. In this respect, this study aimed to evaluate its setting time, radiopacity, antibacterial effect (before setting), and cytocompatibility (after setting) in comparison with other commercially available endodontic materials. The developed material was tentatively named “pozzolan-based bioceramic sealer” (PZBS). Notably, for assessing the antibacterial effect, we compared PZBS with Ca(OH)_2_-based intracanal medicaments. To evaluate the cytocompatibility, we compared PZBS with MTA-based root canal sealers.

## 2. Materials and Methods

### 2.1. Manufacturing of the Material

**Figure 1:**
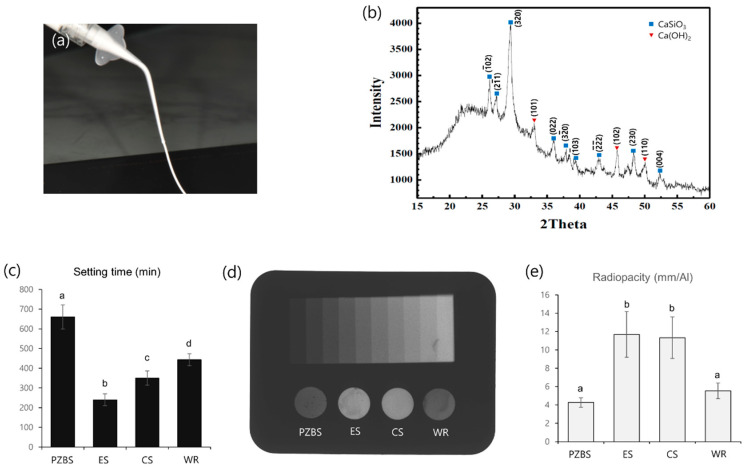
Physical properties of the tested materials: (**a**) The injectable PZBS sealer used in this study. (**b**) X-ray diffraction patterns of PZBS. The hkl index was indicated on the peak. (**c**) The initial setting time of the tested materials. ^abcd^ Different letters represent significant differences between the different materials (*p* < 0.05). (**d**) Radiograph showing the radiopacity of each material and its equivalence to that of the aluminum step wedge. (**e**) Relative radiographic density of each material in comparison with that of a 10-step aluminum step wedge. ^ab^ Different letters represent significant differences between the different materials (*p* < 0.05) PZBS: pozzolan-based bioceramic sealer, ES: EndosealTCS, CS: Ceraseal, WR: Well-Root.

The Ca(OH)_2_ powder (Taekyung BK, Seoul, Korea) was dispersed in dimethyl sulfoxide (DMSO; Amresco, Solon, OH, USA) and then pulverized into 300 nm particles using a nano-particle mill (NPM-0.5L; Nano-intech, Wonju, Korea). Then, the particle size was verified by using a dynamic light scattering particle size analyzer (Zetasizer nano ZS; Malvern Panalytical, Malvern, UK). The silica powder (Brunauer–Emmett–Teller [BET] surface area: 200 m^2^/g) (MFIL-100; Madhu Silica PVT, Gujarat, India) was then mixed with DMSO using a paste mixer (ARE-500; THINKY Corp., Tokyo, Japan) at a revolution speed of 900 rpm and a constant rate of rotation speed (a revolution-to-rotation ratio of approximately 1.0). The two pastes were then mixed so that the molar ratio of calcium to silica was 0.4 using a paste mixer (ARE-500; THINKY Corp.). At this stage, the mixture was used for X-ray diffraction analysis (XRD) to verify whether calcium silicate (CS) was formed by the pozzolan reaction. Zirconium oxide (ZrO_2_) was then added as a radiopaque material. The mixed paste was deposited into an airtight syringe (Schott AG, Mainz, Germany) and used for the experiments (Figure 1a).

### 2.2. Characterization of the Material

#### 2.2.1. XRD Analysis

The crystal phase of the set material was identified using an XRD system (X’pert PRO; PANalytical, Almelo, The Netherlands) and characterized using a database from the International Center for Diffraction Data (Newtown Square, PA, USA). The patterns were obtained under the following conditions: 30.0 mA, 40.0 kV, scan rate: 4°/min, and 10–70°.

#### 2.2.2. Initial Setting Time

The initial setting time of PZBS was evaluated according to the ISO 6876/2012 protocol in comparison with three other CS-based bioceramic root canal sealers, including Endoseal TCS (Maruchi, Wonju, Korea), CeraSeal (Meta Biomed, Cheongju, Korea) and Well-Root (Vericom, Anyang, Korea) (*n* = 7). The setting time was defined as the time at which a one-quarter pound Gilmore indenter failed to leave a definite mark during the initial setting time measurement.

#### 2.2.3. Radiopacity

The radiopacity was measured according to the recommendation of ISO 6876/2012 in comparison with the aforementioned bioceramic sealers. A 99.5% pure aluminum step wedge was constructed with step heights ranging from 1 to 10 mm. Then, the specimens (diameter: 10 mm, thickness: 1 mm) were placed on occlusal X-ray film (Kodak Insight, Rochester, NY, USA) along with the step wedge (*n* = 7). The films were radiated using a Kodak-2200 X-ray machine (Kodak), which was operated at 70 kV, 10 mA, 18 pulses/s, and a focus-sensor distance of 30 cm. The films were converted into digital images and then analyzed using a densitometer (GS-800; Bio-Rad, Hercules, CA, USA) according to the following formula: *y* = *a*ln*x* + *b* (*y*: optical density, *x*: thickness of aluminum, ‘*a*’ and ‘*b*’: coefficients, ln: natural logarithm value).

### 2.3. Antimicrobial Evaluation

#### 2.3.1. Intracanal Biofilm Formation

We prepared standardized bovine root canal specimens as described in a previous study [11]. In brief, we obtained extracted single-rooted bovine central incisors from a slaughterhouse and stored the teeth in 1% sodium hypochlorite (NaOCl) solution for 1 day. Then, we sectioned each tooth horizontally into 5 mm lengths. We enlarged the root canals to 3 mm and then sectioned the specimens vertically into cylindrical halves. We applied a 17% ethylenediaminetetraacetic acid solution to the root canal to remove the smear layer.

*Enterococcus faecalis* (*E. faecalis*; ATCC 29212) was grown aerobically at 37 °C overnight. It was suspended in brain heart infusion media (BHI; Difco Laboratories, Detroit, MI, USA) and standardized spectrophotometrically to 1 × 10^6^ colony-forming units (CFU)/mL. Each sample was placed in a 24-well plate (SPL Lifescience, Pocheon, Korea) containing BHI broth and incubated at 37 °C. After 3 weeks, the specimens of the experimental groups were filled with the Ca(OH)_2_-based materials containing various vehicles: PZBS (DMSO), Ultracal (distilled water) (Ultradent, South Jordan, UT, USA), Calasept Plus (saline) (Nordiska Dental AB, Ängelholm, Sweden), or Calcipex II (polyethylene glycol) (Nippon Shika Yakuhin, Shimonoseki, Japan), and maintained at 37 °C for 10 h. The specimens in the control group were infected, but received no treatment. 

#### 2.3.2. Counting of CFUs

We irrigated the root canals of the experimental groups with 5 mL of sterile water and sonicated the specimens (twice for 10 s at a 20% energy level) to remove the pastes in a water bath (JS Research Inc., Gongju, Korea). We transferred the specimens to a 1.5 mL tube containing 1 mL of sterile water (*n* = 7). An aliquot (0.1 mL) of each specimen was serially diluted. Then, it was plated on BHI agar plates and incubated at 37 °C. After 24 h, CFUs of each sample were counted. 

#### 2.3.3. Confocal Laser Scanning Microscopic (CLSM) Analysis

To investigate the effect of the vehicles on the *E. faecalis* biofilm formed on the bovine root canals, 3-week incubated samples were treated with the Ca(OH)_2_-based medicaments for 10 h and washed with distilled water (*n* = 7). Then, we transferred the specimens to 1.5 mL test tubes with 1 mL of distilled water and sonicated them for 20 s in a water bath (JS Research Inc., Gongju, Korea) to remove the medicaments. We labeled the bacteria remaining on the specimens with the BacLight Bacterial Viability stain (Molecular Probes, Eugene, OR, USA), which was used for staining after sonication for 15 min at room temperature. We performed CLSM imaging of the biofilms using a LSM 510 META microscope (Carl Zeiss, Jena, Germany). We quantified the biofilms based on the confocal stacks using COMSTAT (http://www.comstat.dk (accessed on 10 December 2019)). 

#### 2.3.4. Field-Emission Scanning Electron Microscopy (FE-SEM) Observations

After the treatment of the Ca(OH)_2_ materials, we fixed the biofilms remaining on the specimens with 2.5% glutaraldehyde (Sigma-Aldrich, St. Louis, MO, USA) at 4 °C overnight. We dehydrated the specimens in a graded series of ethanol (25–100%) and a critical point dryer (Leica EM CPD300, GmbH, Vienna, Austria). Then, we observed the samples by FE-SEM (Hitachi, Tokyo, Japan).

### 2.4. Cytocompatibility Evaluation

#### 2.4.1. Preparation of Material Extracts

We placed the tested materials into a mold (1 mm × 5 mm). After setting, the materials were stored in mesenchymal stem cell basal medium (PCS-500-030; ATCC, Manassas, VA, USA) at a ratio of 0.5 cm^2^/mL for 3 days.

#### 2.4.2. Cell Viability Test

We purchased bone marrow derived mesenchymal stem cells (BMMSCs) from ATCC (PCS-500-012). We seeded the cells in 96-well culture plates (SPL Life Sciences) at a density of 3 × 10^3^ cells/well and pre-incubated the cells in growth medium for 24 h (*n* = 7). Then, we treated the cells with the prepared extracts for 24 and 48 h. We measured cell viability using the 3-(4,5-dimethylthiazol-2-yl)-2,5-diphenyltetrazolium bromide (MTT) assay. In brief, we added 200 μL of MTT solution (0.5 mg mL^−1^ in PBS) to each well and incubated the cells for 2 h. Subsequently, we added 200 μL of DMSO to each well. We then shook the plates until the crystals had dissolved, and transferred the solution in each well to a 96-well tissue culture plate. Reduced MTT was then measured spectrophotometrically at 540 nm in a microplate reader (SPECTROstar Nano; BMG Labtech, Ortenberg, Germany).

#### 2.4.3. Cell Migration Assay

To investigate cell migration ability, we performed a scratch wound healing assay. We seeded BMMSCs (1 × 10^6^) in 24-well plates (SPL Life Sciences) and incubated the cells for 24 h (*n* = 7). We made a scratch in the center of the confluent layer of cells using a 200 μL pipette tip. After wounding, we treated the cells with the prepared extracts for 12 h. Images of the scratch area were taken using a phase-contrast microscope (Olympus, Tokyo, Japan). We measured the surface covered by the cells by using an image analysis program (ImageJ; National Institutes of Health, Bethesda, MD, USA). Then, we calculated the area of cell migration into the scratch area using the original scratch area as the reference.

#### 2.4.4. Cell Morphological Observations Using SEM

We seeded the cells at 1 × 10^5^ cells per well on the prepared materials. After a 24 h incubation period, we fixed the materials with 2.5% glutaraldehyde (Sigma-Aldrich) for 2 h. After dehydration, FE-SEM was performed using an SN-3000 system (Hitachi, Tokyo, Japan) operated at 10 kV.

### 2.5. Statistical Analysis

We calculated the sample size using G-Power version 3.1 (University of Düsseldorf, Düsseldorf, Germany). A power analysis with the F test was applied (effect size = 0.8), resulting in the required sample size. We performed the statistical analyses using SPSS version 23 (IBM Corp, Armonk, NY, USA). The data were analyzed using one-way analysis of variance (ANOVA), followed by the Tukey *post hoc* test. A *p*-value less than 0.05 was considered to indicate statistical significance.

## 3. Results

### 3.1. Characterization, Setting Time, and Radiopacity

The formation of C-S-H was verified by the detection of the CS peak (Figure 1b). The initial setting time of PZBS was around 11 h, which was twice as long as the other tested sealers (*p* < 0.05) (Figure 1c). The radiopacity of PZBS was 4.3 mm/Al, which satisfied the ISO criteria, although it was lower than that of EndosealTCS and CeraSeal (*p* < 0.05) (Figure 1d,e).

### 3.2. Antibacterial Effects

**Figure 2:**
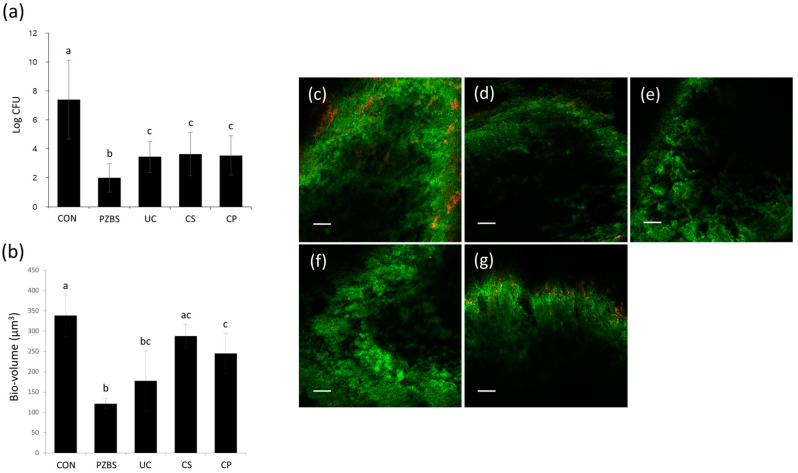
Antibacterial activity of PZBS and the tested intracanal medicaments: (**a**) Colony-forming unit counting. ^abc^ Different letters represent significant differences between the different materials (*p* < 0.05). (**b**) Determination of bio-volume through confocal laser scanning microscopic (CLSM) analysis. ^abc^ Different letters represent significant differences between the different materials (*p* < 0.05). (**c**–**g**) Representative CLSM images of *E. faecalis* biofilms grown on specimens. Scale bar = 100 µm. (**c**) CON; (**d**) PZBS; (**e**) UC; (**f**) CS; (**g**) CP. Different letters represent significant differences between the different materials (*p* < 0.05). CON: control, PZBS: pozzolan-based bioceramic sealer, UC: Ultracal, CS: Calasept, CP: Calcipex II.

**Figure 3:**
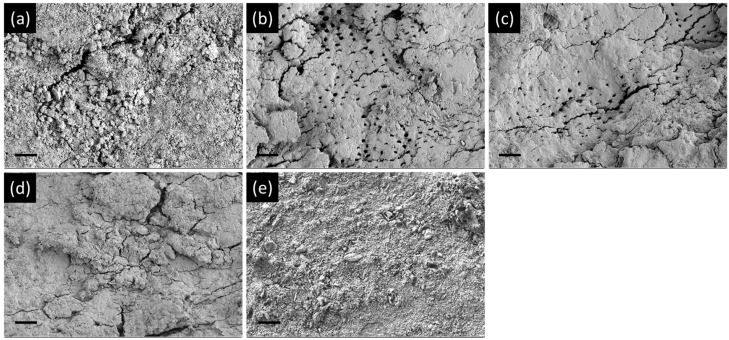
Morphology of *E. faecalis* biofilms formed in the bovine root canal observed by FE-SEM: (**a**) control; (**b**) PZBS; (**c**) Ultracal; (**d**) Calasept; (**e**) Calcipex II. Scale bar = 10 µm.

PZBS showed significantly lower CFU values than Ultracal, Calasept, and Calcipex II (*p* < 0.05) (Figure 2a). PZBS showed a similar bio-volume to that of Ultracal (*p* > 0.05), but a higher bio-volume than those of Calasept and Calcipex II (*p* < 0.05) (Figure 2b–g). The biofilm coverage of the root canal wall was observed under FE-SEM (Figure 3). The control group was characterized by the presence of a thick biofilm layer covering the dentine structure. The NBPS-treated root canal showed more dentinal tubules that were not covered by the biofilms than the other groups.

### 3.3. Cytocompatibility

**Figure 4:**
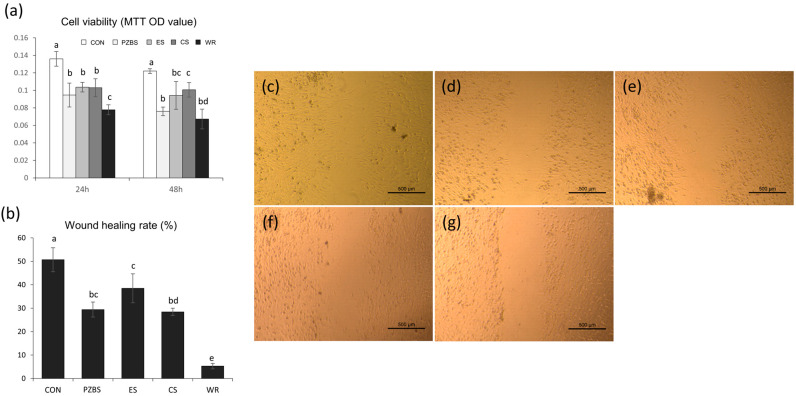
Cytocompatibility of PZBS and the tested root canal sealers: (**a**) Cell viability measured by the MTT assay. ^abcd^ Different letters represent significant differences (*p* < 0.05). (**b**) Wound healing rate measured using the cell migration assay. ^abcde^ Different letters represent significant differences (*p* < 0.05). (**c**–**g**) Representative images of wound healing percentage based on the cell migration assay: (**c**) Control; (**d**) PZBS; (**e**) EndosealTCS; (**f**) Ceraseal; (**g**) Well-Root. CON: control, PZBS: pozzolan-based bioceramic sealer, ES: EndosealTCS, CS: Ceraseal, WR: Well-Root.

**Figure 5:**
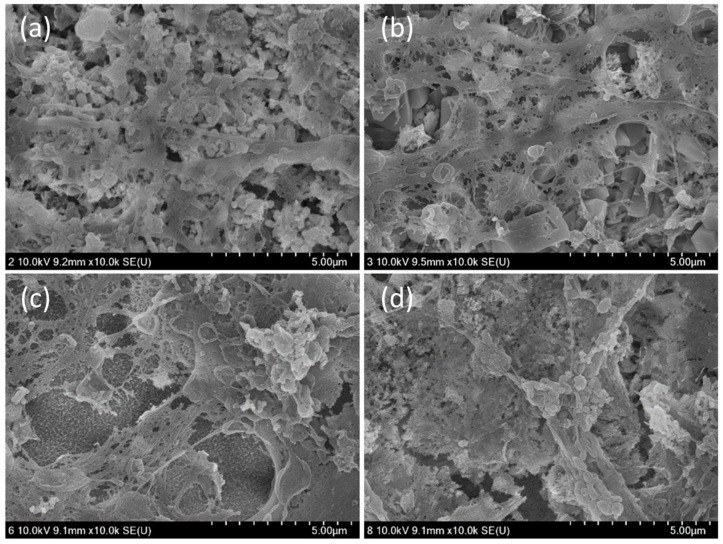
FE-SEM results of direct contact of the cells with each experimental sealer: (**a**) PZBS; (**b**) EndosealTCS; (**c**) Ceraseal; (**d**) Well-Root.

PZBS showed lower cell viability than CeraSeal after 48 h (*p* < 0.05), but comparable viability to the other sealers (*p* > 0.05) (Figure 4a). As shown in Figure 4b–g, the wound healing rate of PZBS assessed using the scratch assay showed similar results to EndosealTCS and CeraSeal (*p* > 0.05), and was higher than that of Well-Root (*p* < 0.05). Furthermore, well-spread and flattened cells in close contact with the surfaces of all the tested materials were observed (Figure 5).

## 4. Discussion

In the present study, firstly, PZBS was manufactured without ZrO_2_ to identify CS because the final wt.% of ZrO_2_ is around 37, and it might interfere with the observation of the CS peak in XRD. The presence of CS in the set PZBS was identified successfully. Moreover, since the pozzolan reaction is a much slower process than Portland cement formation, the Ca(OH)_2_ and silica were nanosized to shorten the setting time. Nevertheless, the initial setting time of PZBS was around 11 h, which was two or three times as long as other tested sealers. However, this may be advantageous because the unreacted Ca(OH)_2_ exerts an antibacterial effect. In the present study, the exposure time of the intracanal medicaments to the biofilm was set to 10 h according to the setting time of PZBS.

In this study, the radiopacity of PZBS was the lowest among the tested sealers, although it satisfied the ISO requirement. The radiopacifier included in the tested sealers is ZrO_2_. The wt.% of ZrO_2_ of other CS-based sealers has not been reported, but it is speculated to be around 50–60% to provide high radiopacity. However, in PZBS, the reaction between Ca(OH)_2_ and silica is critical for producing C-S-H. Consequently, we attempted to reduce the amount of ZrO_2_ to be as low as practical, which decreased the radiopacity.

The antibacterial effect of PZBS was investigated by measuring CFUs and volume of *E. faecalis* biofilms using the standardized bovine root canal model [11]. *E. faecalis* is considered to be a critical bacterial species in endodontics, and it is one of the most prevalent microorganisms isolated from failed root canal cases [12]. The CFU measurements and CLSM analysis revealed that the antibacterial effect of PZBS was higher than that of other groups (Figure 2). It was speculated that the favorable result was due to the nanosized Ca(OH)_2_ in PZBS. It has been reported that nanosized Ca(OH)_2_ shows a stronger antibacterial effect than conventional Ca(OH)_2_, and the high antibacterial activity of nanosized Ca(OH)_2_ could be attributed to its better diffusivity compared to conventional Ca(OH)_2_ [13]. Furthermore, the vehicle used in PZBS was DMSO, which is an important polar aprotic solvent. It has been demonstrated that DMSO exerts an antibacterial effect by inhibiting bacterial pathogenicity and biofilm formation [14,15]. Furthermore, DMSO is frequently used as a vehicle in both in vivo and in vitro experiments since it can dissolve various organic substrates [14,16]. Thus, as shown in the SEM observations (Figure 3b), it can be assumed that the dissolving effect of DMSO might have been responsible for the removal of the biofilm, which is mainly composed of organic substances. Therefore, the DMSO contained in PZBS might be considered to be a supplementary antibacterial agent, which allowed this material to exert a stronger antibacterial effect than other products. In addition, DMSO reduces the microleakage of endodontic sealers, including calcium silicate cement [16,17], which may be an additional advantage of the new material.

Regardless of the type, sealer extrusion may occur unintentionally. Therefore, root canal sealers should be nontoxic after setting to avoid persistent irritation to adjacent periradicular tissue [18]. Therefore, in this study, BMMSCs were selected for cytocompatibility assays since the bone is the major constituent of periradicular tissue, which is in contact with endodontic sealers. Furthermore, several methodologies have been used to evaluate the cytocompatibility of endodontic sealers. Here, we used the MTT assay for the cell viability, the scratch wound healing assay for cell migration, and SEM observations for cell attachment in accordance with previous studies [19,20,21]. In the MTT assay, which is one of the most common methods for measuring the viability of the cells treated with material extract, PZBS showed similar values to those of the other tested sealers except for Ceraseal until 48 h (Figure 4a). The scratch assay, which mimics the extent of migration of cells in vivo during wound healing [22], showed that wound closure occurred for all sealers, with similar rates for PZBS, Endoseal TCS, and Ceraseal (*p* > 0.05) (Figure 4b–g). Furthermore, in the direct contact evaluation using SEM, the cells on the PZBS had attached well and showed cytoplasmic extensions similar to those of other sealers (Figure 5). As a whole, the results indicated that PZBS is cytocompatible after completion of the pozzolan reaction.

## 5. Conclusions

Overall, it is indicated that the newly developed PZBS eventually produced the intended final cementitious product (C-S-H), with an acceptable setting time and radiopacity. Moreover, it showed favorable antibacterial effects and cytocompatibility compared to currently available Ca(OH)_2_-based intracanal medicaments and CS-based root canal sealers, respectively. In conclusion, PZBS has the potential to be used as a root canal sealer that exerts a strong antibacterial effect during the setting reaction.

## Data Availability

Data sharing is not applicable.
